# No symptoms, no problem? an analysis of asymptomatic and symptomatic sexually transmitted infections and the impact on clinical outcomes in a student-run free clinic

**DOI:** 10.1007/s15010-026-02774-2

**Published:** 2026-03-20

**Authors:** Gabriel Alexander Lee, Yuehe Wang, Isabella Gluzman, Gunnar Meyer, Hayley Dunlop, Frances Trezza, Nisha Makkar, Bella Donnelly, Nathan Helsabeck, Courtney DuBois Shihabuddin

**Affiliations:** 1 College of Medicine, The Ohio State University, Columbus, OH USA; 2 Kirksville College of Osteopathic Medicine, A.T. Still University, Kirksville, MO USA; 3 University of Miami, Miller School of Medicine, Miami, FL USA; 4 Department of Obstetrics and Gynecology, University of California, San Francisco, San Francisco, CA USA; 5 The Ohio State University, Columbus, OH USA; 6 College of Nursing, The Ohio State University, Columbus, OH USA

**Keywords:** Sexually transmitted infections, Student-run free clinic, Asymptomatic screening, Health disparities, Underserved populations

## Abstract

**Background:**

Sexually transmitted infections (STIs) remain a major global public health concern, disproportionately affecting uninsured and underserved populations. Student-run free clinics (SRFCs) play a vital role in addressing disparities in STI screening and treatment; however, data describing infection patterns and symptom-based testing outcomes in these settings are limited.

**Methods:**

This IRB-approved retrospective review analyzed 296 patient records from a SRFC between October 2019 and November 2023. Patients who underwent testing for *Chlamydia trachomatis*, *Neisseria gonorrhoeae*, *Treponema pallidum*, *Human Immunodeficiency Virus (HIV)*, and *Human Papilloma Virus (HPV)* were included. Patients were categorized as symptomatic or asymptomatic at the time of testing. Demographic data were collected when available.

**Results:**

Positivity rates were 19.6% for HPV, 7.1% for chlamydia, 7.1% for syphilis, 4.1% for gonorrhea, and 0.5% for HIV. Roughly half of gonorrhea and chlamydia infections occurred in asymptomatic patients. Symptomatic presentation was not significantly associated with positive and negative results. Assigned male at birth (AMAB) patients and younger individuals had higher odds of gonorrhea, while older AMAB patients had higher odds of syphilis.

**Conclusions:**

A substantial proportion of STIs, particularly gonorrhea and chlamydia, occurred in asymptomatic individuals, underscoring the limitations of symptom-based screening. Expanding access to asymptomatic STI testing in SRFCs is essential to reduce missed diagnoses, improve equity, and mitigate long-term sequelae among underserved populations.

## Background

Sexually transmitted infections (STIs) remain a persistent global public health challenge, disproportionately affecting adolescents, young adults, and underserved populations [1]. According to the World Health Organization (WHO), more than 1 million new curable STI cases occur every day, and over 374 million new infections annually are caused by *Chlamydia trachomatis*, *Neisseria gonorrhoeae*, and *Treponema pallidum* [2]. In the United States, the Centers for Disease Control and Prevention (CDC) estimates that 1 in 5 people has an STI at any given time, with young people aged 15–24 accounting for nearly half of all new infections [3].

Despite this high burden, STI prevalence and screening uptake are not evenly distributed across populations. Low-income, uninsured, and marginalized groups face disproportionate risk due to barriers including limited healthcare access, stigma, and lack of routine preventive care [4–6]. Asymptomatic infections are particularly problematic: particularly infections such as chlamydia and gonorrhea often present without symptoms in both patients assigned male at birth (AMAB) and patients assigned female at birth (AFAB), allowing continued transmission and delayed treatment, which can result in pelvic inflammatory disease, infertility, and increased HIV susceptibility [7, 8].

Student-run free clinics (SRFCs) play a growing role in addressing these inequities. Across the United States (US), SRFCs provide low-cost or no-cost care to uninsured and underinsured patients, serving as safety-net clinics while simultaneously training future healthcare providers [9]. These clinics often operate in urban centers and care for populations that are at elevated risk for undiagnosed STIs, yet data on STI screening patterns and outcomes in the SRFC setting remain limited [10].

Understanding STI patterns in SRFCs is critical for optimizing screening strategies, tailoring prevention programs, and reducing missed opportunities for care. Previous studies have shown that targeted STI testing in high-prevalence, underserved settings can be highly cost-effective and improve long-term outcomes [11]. However, given competing demands and limited resources in SRFCs, it is important to determine whether symptom-driven versus routine screening approaches capture the populations most at risk while continuing to remain cost-effective.

Given the growing restrictions on funding for STI testing, especially in low resource settings, and the paucity of literature on STI testing amongst free clinic patient populations, more information about asymptomatic and symptomatic STI testing patterns and outcomes in vulnerable populations is essential to better inform policy [12]. This study aims to describe the relative frequency of STIs in patients presenting to a student-run free clinic, and to compare infection rates among patients undergoing asymptomatic versus symptomatic STI testing. By examining local patterns within this high-risk population, our findings may inform screening protocols, guide resource allocation, and contribute to broader efforts to reduce STI disparities in underserved communities.

## Methods

This study is an IRB approved (2023H0400) retrospective review at a no-cost SRFC associated with a large midwestern tertiary care academic medical center. This SRFC is staffed by medical, nurse practitioner (NP), and physician associate (PA) students who are overseen by licensed physicians, NPs, and PAs. Prior to clinic starting, students are given orientation which includes an overview of services that the clinic provides including STI tests offered at the clinic. The SRFC offers Gonorrhea, Chlamydia, Human Immunodeficiency Virus (HIV), and Syphilis, as well as Human Papilloma Virus (HPV), which was offered as a part of cervical pap smears conducted at the clinic. This study examined 312 patient records who had STI testing from October 2019 to November 2023. Patients with any history of STI testing ordered during the time frame were included in initial review. All STI tests were laboratory-based tests. Sixteen records were excluded either because testing was not performed or no results were reported, resulting in a total of 296 evaluated cases. Patient demographics including sex assigned at birth, gender identity, and race/ethnicity were collected from charts when available. Patient age was calculated from patient date of birth and date of appointment. Chart abstraction was performed by a team of trained researchers, with each chart independently reviewed by at least two reviewers; any discrepancies were resolved by a third reviewer to ensure accuracy and consistency. Symptoms prior to testing were assessed retrospectively and categorized cases into symptomatic or asymptomatic presentation. The electronic medical record (EMR) system used by the clinic has a standardized template for patient encounters which includes heterogeneous list of symptoms that students are expected to ask to patients, and physicians and advanced practice providers (APPs) also review these symptoms in both open and close ended questions with patients if clinically indicated. Due to the nature of the clinic’s EMR, symptoms could not be attributed to a specific STI as many tests involved co-testing of multiple STIs. Data was collected for the following sexually transmitted infections: Gonorrhea, Chlamydia, HIV, Syphilis, and HPV. Site and result were collected if documented.

To examine the difference in STI test results between symptomatic and asymptomatic patients, we conducted two types of analysis and reported on the demographic background of the sample. The first analysis we conducted was to determine whether positive or negative test results occurred at similar rates in symptomatic and asymptomatic patients. To examine this difference, we performed a series of proportional-differences chi square tests. In these tests, we compared both the proportion of negative and positive results between asymptomatic patients and symptomatic patients. In such tests, significant results suggest that there is a difference in the proportion of a given result between asymptomatic and symptomatic patients. Second, we examined if positive test results for chlamydia, gonorrhea, syphilis, or HIV were associated with differences in sex assigned at birth or age by performing a series of logistic regressions. All tests had an alpha set at 0.05 and because the analyses were exploratory, we did not apply a correction for multiple tests. All analyses were conducted in R 4.5.1 [13].

## Results

### Demographics

A majority of the sample were AFAB (67%), had no documented gender (74%), and on average were 33.3 years old. A plurality of the sample identified as Black (42%). Full demographic details are provided in Table 1.

**Table 1:** Demographic Characteristics of Study Sample

	Asymptomatic	Symptomatic	Total
	(*N* = 152)	(*N* = 144)	(*N* = 296)
Age			
Mean (SD)	33.9 (13.0)	32.6 (11.3)	33.3 (12.2)
Median [Min, Max]	30.0 [18.0, 80.0]	29.0 [18.0, 63.0]	30.0 [18.0, 80.0]
Sex at birth			
Female	94 (61.8%)	104 (72.2%)	198 (66.9%)
Male	58 (38.2%)	40 (27.8%)	98 (33.1%)
Race/ethnicity			
Asian	2 (1.3%)	4 (2.8%)	6 (2.0%)
Black	59 (38.8%)	64 (44.4%)	123 (41.6%)
Latino	10 (6.6%)	10 (6.9%)	20 (6.8%)
Middle East/N African	1 (0.7%)	0 (0%)	1 (0.3%)
Two or more	8 (5.3%)	9 (6.3%)	17 (5.7%)
Unknown	44 (28.9%)	42 (29.2%)	86 (29.1%)
White	28 (18.4%)	15 (10.4%)	43 (14.5%)

### Differences in testing and results

Table 2 provides rates of tests by sex assigned at birth and symptom status. Across all tests regardless of symptom status females were tested at a higher rate than males and this was true for both symptomatic and asymptomatic patients.

**Table 2:** Rates of Testing by Sex Assigned at Birth and Symptom Status

	Asymptomatic		Symptomatic		Total	
	Female	Male	Female	Male	Female	Male
	(*N* = 94)	(*N* = 58)	(*N* = 104)	(*N* = 40)	(*N* = 198)	(*N* = 98)
Gonorrhea						
No	21 (22.3%)	23 (39.7%)	18 (17.3%)	8 (20.0%)	39 (19.7%)	31 (31.6%)
Yes	73 (77.7%)	35 (60.3%)	86 (82.7%)	32 (80.0%)	159 (80.3%)	67 (68.4%)
Chlamydia						
No	21 (22.3%)	23 (39.7%)	18 (17.3%)	7 (17.5%)	39 (19.7%)	30 (30.6%)
Yes	73 (77.7%)	35 (60.3%)	86 (82.7%)	33 (82.5%)	159 (80.3%)	68 (69.4%)
HIV						
No	20 (21.3%)	15 (25.9%)	42 (40.4%)	22 (55.0%)	62 (31.3%)	37 (37.8%)
Yes	74 (78.7%)	43 (74.1%)	62 (59.6%)	18 (45.0%)	136 (68.7%)	61 (62.2%)
Syphilis						
No	34 (36.2%)	23 (39.7%)	39 (37.5%)	17 (42.5%)	73 (36.9%)	40 (40.8%)
Yes	60 (63.8%)	35 (60.3%)	65 (62.5%)	23 (57.5%)	125 (63.1%)	58 (59.2%)
HPV						
No	46 (48.9%)	58 (100%)	55 (52.9%)	40 (100%)	101 (51.0%)	98 (100%)
Yes	48 (51.1%)	0 (0%)	49 (47.1%)	0 (0%)	97 (49.0%)	0 (0%)

In the comparison of proportions of negative and positive test results between symptomatic and asymptomatic patients, we found no differences in gonorrhea, chlamydia, syphilis or HPV. We did find a significantly higher proportion of negative results for symptomatic patients for HIV (p = 0.005). Full results are available in Table 3. Importantly, Table 3 notes that the results of some of the comparisons of positive test results should be interpreted with caution due to the small number of positive results available in the sample.

**Table 3:** Comparison of proportions between symptomatic and asymptomatic tests

	Asymptomatic	Symptomatic	Total	*χ*^2^ (df = 1)	*p*	95% CI	
Gonorrhea	(*n* = 108)	(*n* = 118)	(*n* = 226)				
Negative	88 (81%)	101 (86%)	189 (84%)	1.524	0.217	− 0.175	0.037
Positive*	4 (3%)	4 (3%)	8 (4%)	0.000	1.000	− 0.490	0.490
Inconclusive/unknown	16 (11%)	13 (11%)	29 (13%)				
Chlamydia	(*n* = 108)	(*n* = 119)	(*n* = 227)				
Negative	86 (80%)	97 (82%)	183 (81%)	1.093	0.296	− 0.168	0.048
Positive	6 (6%)	8 (7%)	14 (6%)	0.143	0.706	− 0.581	0.295
Inconclusive / unknown	16 (11%)	14 (12%)	30 (13%)				
HIV	(*n* = 117)	(*n* = 80)	(*n* = 197)				
Negative	106 (91%)	78 (98%)	184 (93%)	7.924	0.005	0.046	0.259
Positive*	1 (< 1%)	0 (0%)	1 (< 1%)	0.000	1.000	0.000	1.000
Inconclusive/unknown	10 (9%)	2 (3%)	12 (6%)				
Syphilis	(*n* = 95)	(*n* = 88)	(*n* = 183)				
Negative	85 (89%)	78 (89%)	163 (89%)	0.442	0.506	− 0.072	0.158
Positive	4 (4%)	6 (7%)	10 (6%)	0.200	0.655	− 0.729	0.329
Inconclusive/unknown	6 (6%)	4 (5%)	10 (6%)				
HPV	(*n* = 48)	(*n* = 49)	(*n* = 97)				
Negative	20 (42%)	17 (35%)	37 (38%)	0.216	0.642	0.541	0.459
Positive*	6 (13%)	3 (6%)	9 (9%)	0.889	0.346	− 0.213	0.880
Inconclusive/unknown	22 (46%)	29 (59%)	51 (53%)				

The * indicates that results should be interpreted with caution as chi-squared approximation may be incorrect due to small sample size

### Differences associated with sex and age

In our sample, we found that patients that were AMAB were significantly more likely to test positive for gonorrhea (p = 0.017) and syphilis (p = 0.016). Additionally, we found that for every year of increased age there were higher odds for testing positive for syphilis (p = 0.027) and for every year of decreased age there were lower odds associated with testing positive for gonorrhea (p = 0.038). Full results are available on Tables 4 and 5.

**Table 4:** Sex and age characteristics of STIs and associated symptom presentation and outcomes

	Asymptomatic		Symptomatic		Total	
	Negative	Positive	Negative	Positive	Negative	Positive
Gonorrhea	(*N* = 88)	(*N* = 4)	(*N* = 101)	(*N* = 4)	(*N* = 189)	(*N* = 8)
Sex						
Female	59 (67.0%)	1 (25.0%)	75 (74.3%)	1 (25.0%)	134 (70.9%)	2 (25.0%)
Male	29 (33.0%)	3 (75.0%)	26 (25.7%)	3 (75.0%)	55 (29.1%)	6 (75.0%)
Age						
Mean (SD)	32.7 (11.0)	21.0 (1.83)	33.4 (11.6)	25.8 (7.54)	33.1 (11.3)	23.4 (5.68)
Median [Min, Max]	29.5 [18.0, 67.0]	21.0 [19.0, 23.0]	30.0 [18.0, 63.0]	22.5 [21.0, 37.0]	30.0 [18.0, 67.0]	22.0 [19.0, 37.0]
Chlamydia	(*N* = 86)	(*N* = 6)	(*N* = 97)	(*N* = 8)	(*N* = 183)	(*N* = 14)
Sex						
Female	55 (64.0%)	5 (83.3%)	69 (71.1%)	7 (87.5%)	124 (67.8%)	12 (85.7%)
Male	31 (36.0%)	1 (16.7%)	28 (28.9%)	1 (12.5%)	59 (32.2%)	2 (14.3%)
Age						
Mean (SD)	32.9 (11.0)	22.0 (3.52)	33.4 (11.8)	30.5 (8.40)	33.2 (11.4)	26.9 (7.86)
Median [Min, Max]	30.0 [18.0, 67.0]	21.5 [18.0, 28.0]	31.0 [18.0, 63.0]	27.5 [23.0, 47.0]	30.0 [18.0, 67.0]	25.5 [18.0, 47.0]
HIV	(*N* = 106)	(*N* = 1)	(*N* = 78)	(*N* = 0)	(*N* = 184)	(*N* = 1)
Sex						
Female	69 (65.1%)	0 (0%)	60 (76.9%)		129 (70.1%)	0 (0%)
Male	37 (34.9%)	1 (100%)	18 (23.1%)		55 (29.9%)	1 (100%)
Age						
Mean (SD)	33.6 (13.3)	47.0 (NA)	31.7 (10.8)		32.8 (12.3)	47.0 (NA)
Median [Min, Max]	30.0 [18.0, 80.0]	47.0 [47.0, 47.0]	28.0 [18.0, 63.0]		29.0 [18.0, 80.0]	47.0 [47.0, 47.0]
Syphilis	(*N* = 85)	(*N* = 4)	(*N* = 78)	(*N* = 6)	(*N* = 163)	(*N* = 10)
Sex						
Female	57 (67.1%)	0 (0%)	58 (74.4%)	3 (50.0%)	115 (70.6%)	3 (30.0%)
Male	28 (32.9%)	4 (100%)	20 (25.6%)	3 (50.0%)	48 (29.4%)	7 (70.0%)
Age						
Mean (SD)	31.9 (10.9)	40.0 (11.4)	31.6 (11.2)	40.2 (8.30)	31.7 (11.0)	40.1 (9.04)
Median [Min, Max]	28.0 [18.0, 67.0]	37.5 [29.0, 56.0]	27.5 [18.0, 63.0]	39.0 [29.0, 54.0]	28.0 [18.0, 67.0]	37.5 [29.0, 56.0]
HPV	(*N* = 20)	(*N* = 6)	(*N* = 17)	(*N* = 3)	(*N* = 37)	(*N* = 9)
Sex						
Female	20 (100%)	6 (100%)	17 (100%)	3 (100%)	37 (100%)	9 (100%)
Age						
Mean (SD)	42.5 (9.20)	40.7 (11.2)	38.7 (9.10)	38.3 (8.62)	40.7 (9.22)	39.9 (9.94)
Median [Min, Max]	43.5 [22.0, 56.0]	40.0 [30.0, 60.0]	40.0 [22.0, 57.0]	40.0 [29.0, 46.0]	41.0 [22.0, 57.0]	40.0 [29.0, 60.0]

**Table 5:** Odds ratio results for gonorrhea, chlamydia, and syphilis

Positive result	Term	Odd ratios	Standard error	*p* value
Chlamydia	(Intercept)	0.097 [0.051,0.168]	0.302	0
	male	0.35 [0.053,1.338]	0.78	0.179
Gonorrhea	(Intercept)	0.015 [0.002, 0.047]	0.712	0
	male	7.309 [1.627, 50.946]	0.832	0.017
Syphilis	(Intercept)	0.026 [0.006, 0.069]	0.585	0
	male	5.59 [1.487, 26.78]	0.711	0.016
Chlamydia	(Intercept)	0.557 [0.087, 4.397]	0.984	0.551
	age	0.935 [0.865, 0.993]	0.035	0.054
Gonorrhea	(Intercept)	3.063 [0.133, 214.656]	1.849	0.545
	age	0.853 [0.706, 0.96]	0.077	0.038
Syphilis	(Intercept)	0.008 [0.001, 0.056]	1.08	0
	age	1.059 [1.006, 1.117]	0.026	0.027

## Discussion

This study aimed to examine the relationship between symptomatic presentation, testing outcomes, and demographic factors for a wide range of STIs. Amongst all STIs analyzed, there was no overarching consistency in both the proportion of asymptomatic presentations and the demographics associated with positive and negative results.

Gonorrhea and chlamydia trended similarly, with 4.1% and 7.1% positivity rates, respectively, which were higher than the 2024 United States national positivity rates of 1.6% and 4.5% for gonorrhea and chlamydia, respectively [14]. Amongst the gonorrhea-positive patients, half presented without symptoms, which is consistent with previous literature demonstrating the variability in symptom presentation for gonorrhea [15, 16]. Younger AMAB patients demonstrated a higher odds of testing positive for gonorrhea, consistent with current epidemiological patterns demonstrating higher positivity rates amongst younger adults in the United States and supporting continued routine screening amongst these populations [16]. Chlamydia demonstrated similar trends with slightly more than half of the positive patients presenting with symptoms, suggesting that symptom-based testing alone would fail to catch some of these cases. The lack of statistical difference between symptomatic and asymptomatic STI testing outcomes for gonorrhea and chlamydia reaffirms the current recommendations to screen individuals who are sexually active individuals under the age of 25 and higher risk populations regardless of symptom presentation [17].

Syphilis demonstrated a 7.1% positivity rate with older AMAB patients having a higher odds of testing positive. This was lower than the rate of reported cases (0.056%) in 2024, which could be associated with higher prevalence of STIs in communities of lower socioeconomic status [14, 18]. The lack of significant association between symptom presentation and test positivity demonstrates the importance of continued testing for syphilis in high-risk groups and maintaining a low threshold for testing in the setting of high clinical suspicion to avoid life threatening complications such as neurosyphilis.

HPV testing is not regularly performed outside of cervical cancer screenings and was performed when indicated with routine cervical cancer screenings or pap smears. There was no association between symptoms and testing outcomes, consistent with HPV often not presenting with any symptoms [19]. These data reinforce the value of routine cervical cancer screenings with HPV co-testing when appropriate and HPV vaccination efforts as important and effective public health measures.

Overall, these data suggest that symptom-based testing is insufficient to identify many STIs, particularly gonorrhea, chlamydia, and syphilis, amongst our largely uninsured population. Given the sequelae of untreated STIs and the limited contact that uninsured or underinsured patients may have with the healthcare system, availability of free STI testing for patients with and without STI symptoms is essential [20]. The current CDC guidelines generally promote asymptomatic STI testing, especially for high-risk populations [17]. This study supports the notion that STI testing needs to be widely accessible to patients of all socioeconomic backgrounds. SRFCs are an important provider of free STI testing and are uniquely positioned to provide free asymptomatic STI screens to uninsured or underinsured patients while they are being evaluated and treated for STIs and other health conditions. These data elucidate the need for availability of additional funding for SRFCs and other low-cost clinics to provide STI screenings to remove barriers to vital health care and improve the overall well-being of under-resourced patients. Furthermore, given the high rates of positive tests that present without symptoms, increased patient education could be important at curbing the spread of STIs, especially those that often present asymptomatically. Patients of lower socioeconomic status are associated with lower levels of health literacy [21]. Improving health literacy has been demonstrated to be effective at decreasing high-risk behaviors and overall incidence of STIs [22].

Additional research is necessary to validate these findings in a larger, multicenter cohort and incorporate additional behavioral and social determinants of health data, such as sexual practices, partner networks, and Pre-Exposure Prophylaxis (PrEP) usage to better understand risk associated with STIs in this population. Future work should include longitudinal analyses that capture reinfection and recurrent trends amongst uninsured and underinsured populations. Ultimately, this study demonstrates the importance of the availability of STI testing for uninsured and underinsured populations, regardless of symptoms, to reduce the overall incidence of STIs in the United States.

### Strengths and limitations

The main strength of this paper is that it is one of the first of its kind to evaluate the STI testing outcomes of predominantly uninsured patients at a SRFC. Most studies focus on large health care centers where a large majority of patients are insured. This study contributes to a necessary gap in knowledge about the most vulnerable patients in the United States. For that same reason, the study is limited in its size and location. The study was conducted at a single site over a 4-year period, utilizing an EMR system that did not always have consistently reported demographic data. Additionally, documentation was conducted largely by student learners, which could contribute to some inconsistencies in documentation. This reduces its generalizability to other clinics; however, it suggests the importance of increasing the number of studies of this type at other free clinics both nationally and internationally to add to the findings produced by this study. The test results that were analyzed in this study were all treated as individual cases due to limitations with documentation in our EMR system. The act of collecting samples from patients varied between patients based on student learner comfort. This could have resulted in insufficient samples collected; however, samples that were insufficient were excluded from analysis as they did not produce a result and licensed providers were always with student learners. Furthermore, the clinic’s EMR did not make distinctions for the individual collecting the sample, making it difficult to discern from review. A follow up study at this SRFC may be warranted because of recent changes in EMR practices, increasing the consistency of demographic data, including sexual orientation and gender identity, and the establishment of our new LGBTQIA2 + and PrEP clinics, which provides asymptomatic STI testing [23].

## Conclusion

Asymptomatic STI testing continues to play an important role in preventative health and this study corroborates this conclusion in the context of uninsured patient populations. SRFCs continue to play a pivotal role in the healthcare of uninsured patients and must continue to balance limited financial constraints to provide comprehensive care that patients would not otherwise be able to obtain. While STI testing is important, it is equally important to recognize the unique situations of individual free clinics and to maximize services that will best serve the population they see. More research is necessary to support the findings of this paper; however, we hope that this work can highlight the importance of STI testing in free clinic settings and the importance of SRFCs in contributing to strides in preventive public health interventions.

## Data Availability

The data that support the findings of this study are not openly available due to reasons of sensitivity and are available from the corresponding author upon reasonable request.
